# Fever in the Returning Traveler: A Practical Overview for Initial Management and Assessment in the ED

**DOI:** 10.3390/jcm15124733

**Published:** 2026-06-18

**Authors:** Liesbeth Van Dessel, Peter Vanbrabant, Liesbet Henckaerts, Marc Sabbe

**Affiliations:** 1 Department of Emergency Medicine, University Hospitals Leuven, 3000 Leuven, Belgium; liemarc.sabbe@uzleuven.be; 2 Department of General Internal Medicine, University Hospitals Leuven, 3000 Leuven, Belgium; peter.vanbrabant@uzleuven.be; 3 Department of Microbiology and Transplantation, University Hospitals Leuven, 3000 Leuven, Belgium; liesbet.henckaerts@uzleuven.be

**Keywords:** fever, returning traveler, emergency department, travel-related infections

## Abstract

**Background**: International travel has increased over recent decades, leading to a rise in the number of patients presenting to emergency departments (EDs) with fever after returning from abroad. Evaluating fever in returning travelers is challenging because the differential diagnosis is broad, and exposure to tropical diseases limited, among most ED clinicians. **Objective**: This article aims to provide a practical overview of the most common travel-related causes of fever. The tool is intended to support targeted diagnostics and timely treatment and/or timely specialist referral, while emphasizing that non-travel-related infections must also be considered. **Methods**: We created a clinical summary of the most common causes of fever in returning travelers based on epidemiology, incubation periods, clinical features, and diagnostic approaches. A practical overview was created to aid ED clinicians in evaluating stable patients, incorporating travel history, exposure risks, and key clinical findings. **Results**: Malaria, dengue and typhoid fever are among the most common diagnoses in travelers returning from abroad, excluding non-travel-related diseases. These conditions share overlapping symptoms. Diagnosis relies on clinician awareness and a combination of exposure history, clinical evaluation, and targeted laboratory testing. Treatment depends on the causative pathogen and disease severity, but often requires early empiric therapy and supportive care. **Conclusions**: This article presents a systematic, pragmatic approach to the evaluation of fever in the returning traveler. This overview is designed to help ED clinicians recognize and make appropriate initial management and referral decisions when assessing a stable traveler. Nevertheless, we recommend specialist advice for most cases.

## 1. Introduction

The number of people traveling internationally has increased steadily over the past several decades, and in 2025, 52 billion people are expected to cross international borders [1]. As global mobility has expanded, so too has the number of travelers with post-travel fever who are seen in primary-care and emergency-room settings. This poses a diagnostic challenge, particularly in settings with limited exposure to tropical diseases [2].

Among travelers, those visiting friends and relatives (VFR travelers) are at highest risk of acquiring travel-related infections, as they are less likely to seek pre-travel care and use prophylactic measures, and have an increased exposure to rural areas, food and water. Nevertheless, most cases of post-travel fever remain ultimately caused by common, non-travel-related infections such as influenza, COVID-19, and other (viral) pathogens [3,4].

A detailed travel and exposure history is essential to identify potential tropical infections and is the primary diagnostic tool to select directed laboratory testing. In the emergency department (ED), however, differentiating between self-limiting febrile illnesses and potentially severe infections (whether or not imported) remains difficult. This is compounded by a general lack of familiarity with tropical diseases among ED clinicians, due to limited training and exposure [2,3,5].

Here, we present a non-comprehensive review of travel-associated infections that must be considered in the case of fever. We provide a summary to guide clinicians through the initial evaluation, management and referral process. For returning travelers, better recognition of febrile illness in the E.D. setting can facilitate both appropriate triage and timely referral to specialist care, if needed. The overview was designed to be applied outside of the ICU, in patients who did not need immediate intensive care.

## 2. Materials and Methods

With this narrative review, we aimed to summarize the most common causes of post-travel fever and created a comprehensive overview. A literature search was conducted using PubMed to identify relevant studies published in the last 10 years.

The search strategy included combinations of the following keywords: “fever in returning travelers,” “tropical fever,” “imported infections,” and “travel-related infectious diseases.” Articles were screened based on their relevance with respect to epidemiology, clinical presentation, and prevalence of infectious causes of fever acquired in tropical settings.

Priority was given to studies describing conditions most frequently encountered in clinical practice. Additional articles were identified through manual screening of reference lists of selected publications.

As this study was designed as a narrative review, no formal systematic review protocol was followed, and no structured quality assessment or meta-analysis was performed.

## 3. Results

### 3.1. Assessment of Travel History, Itinerary and Exposure

When a patient presents in the emergency department with fever after travel to the tropics or subtropics, thorough history taking is the essential first step in establishing a differential diagnosis. A detailed travel history is important, not only the overall itinerary but also the exact dates and specific areas (both urban and rural) that were visited [2,3,5], and activities undertaken. 

For example, Suriname in South America is considered essentially malaria-free in most cities, but sporadic cases are seen in remote, rural areas. Only travel to remote border zones significantly increases malaria risk; absence of such travel renders malaria exceedingly unlikely [6,7,8]. Also, the duration of the incubation period will often help narrow the differential diagnosis. When the incubation period is short (e.g., <14 days), one of the first considerations is malaria, but arboviral infections, dengue, leptospirosis, and enteric fever are also possible. Conversely, a single overnight stop in a malaria-endemic region followed by fever onset the next day makes malaria biologically implausible, given that the incubation period for *Plasmodium falciparum* is typically longer than seven days [9].

Equally important is a systematic exposure history: freshwater exposures (including rivers, lakes, and flooded streets or aerosol-generating devices) raise suspicion of leptospirosis or schistosomiasis. Sexual contacts indicate risk for acute HIV, other sexually transmitted infections (STIs), or viral hepatitis. Exposure to animals raises suspicion of brucellosis or Q fever. The purpose of travel should also be recorded, as VFR travelers are often at greater risk because of increased exposure to rural areas, local food and water, and lower rates of prophylaxis and vaccination [2,3,5,9].

Clinicians also need to ask about pre-travel consultations, vaccination status, chemoprophylaxis compliance (e.g., antimalarials), and personal protective measures (e.g., use of mosquito nets, insect repellents, and safe food and water precautions).

After establishing that the patient is hemodynamically stable and not in shock, we proceed to a complete physical examination: we note the presence or absence of skin lesions (e.g., rashes, eschars or petechiae), lymphadenopathy, hepatosplenomegaly, and pulmonary, gastrointestinal, or neurologic abnormalities. Such findings help refine differential diagnoses, especially in cases of undifferentiated fever [2].

Given that many travel-associated infections require time-consuming diagnostics, careful history-taking and clinical assessment are essential to guide appropriate testing and empirical therapy.

We propose an initial emergency department approach to fever in the returning traveler that begins with a rapid assessment of hemodynamic stability. This should be followed by a comprehensive travel history, including dates of departure and return, regions visited, and potential exposures to risk factors such as freshwater contact, insect bites, food and water sources, and animal contact.

A thorough clinical examination is an essential component of the initial evaluation and can significantly aid in narrowing the differential diagnosis. Particular attention should be paid to vital signs, skin findings (e.g., rash, eschars, petechiae), lymphadenopathy, hepatosplenomegaly, and signs of organ dysfunction. Certain clinical patterns—such as jaundice, hemorrhagic manifestations, or neurological abnormalities—may point toward specific infectious etiologies and guide further diagnostic testing.

Subsequently, we provide a concise review of several common tropical diseases associated with fever. This overview is not exhaustive, but highlights key conditions relevant to clinical practice. For each disease, we outline the typical clinical presentation, recommended diagnostic approach, and appropriate laboratory investigations.

As part of the initial assessment, we recommend obtaining broad laboratory investigations, including blood cultures and routine hematological and biochemical analyses. These tests should then be tailored based on the patient’s travel history and suspected regional pathogens. For example, in travelers returning from malaria-endemic areas, prompt microscopic examination of thick and thin blood smears—or equivalent rapid diagnostic testing—should be performed to exclude or confirm malaria.

The following is a brief review of several common tropical diseases that result in fever, but they do not reflect all febrile illnesses. For each disease we provide a brief description of the clinical presentation: the diagnostic approach, laboratory tools, the key epidemiologic features, such as geography, vector or mode of exposure, and incidence.

Q fever and brucellosis are also listed. Although these are rare causes of fever in the returning traveler, they should be considered when risk factors such as animal exposure are present [10].

### 3.2. Short Incubation Time (<14 Days)

#### 3.2.1. Malaria

Malaria is endemic in large areas of sub-Saharan Africa, South-East Asia, Eastern Mediterranean, Western Pacific, Latin America and parts of the Caribbean. Transmission occurs after the bite of an infected female Anopheles mosquito. *Plasmodium falciparum* and *Plasmodium vivax* are, worldwide, the most prevalent of the five types to cause malaria in humans [11]. *P. falciparum* causes the most dangerous and fatal forms of malaria and is mostly found in Africa, while *P. vivax* causes a relapsing form and is more frequently encountered in South-East Asia and Central America [3].

Clinical presentation is typically nonspecific, with symptoms including fever, headache, malaise, myalgia, fatigue, diaphoresis, gastrointestinal symptoms, and dry cough. Diagnosis can be especially challenging in children and they are at increased risk of severe disease, particularly anemia and cerebral malaria. Severe malaria, most commonly due to *P. falciparum*, can be associated with metabolic acidosis, hypoglycemia, renal failure, respiratory distress, shock, and hyperparasitemia [10,11,12,13].

The diagnosis is usually made by microscopy using thick and thin blood smears, ideally obtained during febrile peaks and repeated up to three times at 12–24 h intervals if suspicion persists [12]. Parasitemia should be monitored daily during treatment, until clearance. Rapid diagnostic tests may be useful when microscopy is unavailable, but have lower sensitivity at low parasitemia and cannot reliably differentiate species.

Urgent treatment is essential to prevent morbidity and mortality. Confirmed and uncomplicated non-falciparum malaria may be managed in an outpatient setting, but suspected or confirmed *P. falciparum* malaria should be treated as a medical emergency. Treatment of non-falciparum infection consists of either chloroquine or artemisinin-based combination therapy (ACT) if there is chloroquine resistance. Uncomplicated *P. falciparum* cases are preferentially treated with ACT or atovaquone–proguanil, with quinine-based regimens as an alternative, and severe disease is initially treated with intravenous artesunate. *P. vivax* or *P. ovale* infections have dormant liver stages (hypnozoites) that require additional treatment directed against the liver stages, to prevent relapses [3,14,15,16].

#### 3.2.2. Dengue

Dengue virus (DENV), a flavivirus transmitted by Aedes mosquitoes, is endemic in the tropical and subtropical regions of Southeast Asia, Central Asia, the Americas and Central Africa [6,17,18].

Dengue presents across a broad clinical spectrum, ranging from asymptomatic infection with a mild flu-like illness to dengue hemorrhagic fever (DHF) and its most severe form, dengue shock syndrome (DSS). Initial clinical manifestations include high-grade fever and severe myalgia and arthralgia (hence the colloquial term “break-bone fever”). In the minority of cases, this is followed by a critical phase, with severe dengue manifesting as plasma leakage, hemorrhagic manifestations, and multi-organ dysfunction [19,20,21,22,23,24].

Retro-orbital pain and headache may be more prominent in dengue than in chikungunya.

Diagnosis is established by PCR or serology.

There is no specific antiviral treatment for dengue, so treatment is mainly focused on supportive care. Clinicians often face the challenge of distinguishing dengue from other febrile illnesses that are prevalent in the same areas, such as chikungunya and malaria [2,3,19]. Furthermore, the administration of nonsteroidal anti-inflammatory drugs (NSAIDs), such as ibuprofen or aspirin, is contraindicated in cases of dengue but is often standard treatment for chikungunya [21].

Risk factors for severe dengue include young age, patients with (several) comorbidities, and previous dengue infection [22,23]. A secondary infection with a different DENV serotype can lead to more severe disease, a phenomenon attributed to antibody-dependent enhancement (ADE) [4,5]. A live-attenuated tetravalent vaccine is therefore only approved for individuals with confirmed prior dengue infection, as vaccination of seronegative individuals may also increase the risk of severe dengue upon subsequent infection. For this reason, dengue serology is recommended as part of the diagnostic work-up, even in the absence of immediate therapeutic consequences, as it provides important information for future vaccination eligibility and risk stratification.

#### 3.2.3. Hemorrhagic Fever

Viral hemorrhagic fevers include infections caused by viruses in several families (*Filoviridae*, *Arenaviridae*, *Flaviviridae*, *Nairoviridae*, *Phenuiviridae* and *Hantaviridae*). Transmission is through arthropod vectors (e.g., mosquitoes and ticks for yellow fever, dengue fever, Rift Valley fever and Crimean–Congo hemorrhagic fever), through contact with infected rodents, which are the reservoirs (e.g., for Lassa fever and hantavirus infections), or through human contact with body fluids and respiratory droplets (e.g., for Ebola virus disease, Lassa fever, Marburg virus disease) [25,26].

All the above mentioned infections have similar clinical features, including an acute febrile response and increased vascular permeability, and, in severe forms, hemorrhagic manifestations and multiorgan dysfunction [27,28,29]. The ease with which the disease spreads and the severity of the infection present a considerable challenge to public health teams, who must rapidly enhance their prevention and control measures to prevent transmission. Such patients are attended by health care personnel using personal protective equipment in specialized high-level isolation units with strict infection-control procedures [26,30].

In general, a patient is suspected of having viral hemorrhagic fever if he or she has a febrile illness that develops within 3 weeks after exposure to a potential source in an endemic area and is associated with at least two hemorrhagic symptoms (e.g., petechiae, purpura, bleeding from the gums, bleeding from injection sites, or blood in the stool). Leukopenia, thrombocytopenia, elevated transaminases and coagulation abnormalities are common laboratory findings. Symptomatic travelers returning from areas with active outbreaks should be evaluated urgently and with care, in a specialized center [27,28,29].

Diagnosis, based on epidemiological exposure history, clinical findings and laboratory testing, should include strict attention to infection control measures. Laboratory confirmation is mainly by assays, such as PCR, which are the diagnostic standard; serologic tests are supportive in later stages of the disease.

Treatment is mainly supportive, and should be initiated as soon as possible. Specific antiviral therapies are available for only a few of these infections—for example, ribavirin for some arenavirus and bunyavirus infections and monoclonal antibodies for Ebola virus disease. Vaccination is also available for some hemorrhagic fevers known to be caused by viruses, such as yellow fever and Ebola and Marburg viruses [29].

#### 3.2.4. Chikungunya

Chikungunya virus (CHIKV) is an arbovirus infection primarily transmitted by *Aedes aegypti* and *Aedes albopictus.* The same vectors are endemic in regions where dengue circulates as well [31]. The disease has an incubation period of approximately 3 to 7 days [32].

Clinically, it mimics dengue, with abrupt onset of high fever, myalgia, generalized malaise, rash, and sometimes gastrointestinal symptoms such as nausea, vomiting, or diarrhea. A distinguishing feature is severe, bilateral, often symmetric, polyarthralgia or arthritis, especially involving small joints of the hands and feet, wrists, ankles and knees, which may take the form of a disabling polyarthritis in the acute phase and in some patients persist for weeks to months [32,33].

Management is typically outpatient and supportive, relying on adequate analgesia with NSAIDs or, if needed, opioids. Despite acute treatment, persistent and debilitating arthritis necessitating immunosuppressive therapy may occur in a later stage [34].

Severe complications such as encephalitis, Guillain–Barré syndrome, or severe sepsis can arise, particularly in neonates, elderly persons, or immunocompromised individuals [35].

Probable infection is defined as compatible clinical presentation plus at least one positive serological test, whereas definitive (certain) diagnosis requires clinical criteria together with serology (e.g., IgM or IgG) or RT-PCR confirmation [3,33].

#### 3.2.5. Rickettsiosis

Rickettsiosis is caused by Gram-negative intracellular bacteria of the genus *Rickettsia.* These bacteria are transmitted by arthropod vectors, such as ticks, mites and fleas. The main rickettsial syndromes are he following: the Spotted Fever Group (SFG), which is found worldwide in regions including North America (e.g., Rocky Mountain spotted fever), the Mediterranean, Africa, the Middle East, and northern parts of Australia; the Scrub Typhus Group (STG), found primarily in Southeast Asia and northern Australia; and the Typhus Group (TG), seen predominantly in urban areas of Southeast Asia [36]. Patients with rickettsiosis usually present with fever and an eschar at the site of bacterial inoculation (more common in STG and SFG), along with a maculopapular rash and gastrointestinal symptoms (e.g., nausea, vomiting, or diarrhea) [37,38].

In Southeast Asia, rickettsiosis is the second most common organism causing non-malarial febrile illnesses in patients, only exceeded by dengue fever. It is difficult to distinguish the illness clinically from dengue in the early stages, and serological confirmation may take 10 to 14 days. Empirical doxycycline (or azithromycin for pregnant patients of children) for patients with acute undifferentiated fever returning from an endemic region may therefore be considered, and is likely to improve outcomes [39].

#### 3.2.6. Non-Travel-Related Infections

When patients present with fever after travel from tropical regions, clinicians should also consider non–travel-related infections. Common etiologies remain more likely than exotic tropical pathogens. Thorough history taking and clinical examination should therefore inform initial diagnostic testing and empiric management, while maintaining vigilance for less frequent imported diseases [3,4].

### 3.3. Medium Incubation Time (14–30 Days)

#### 3.3.1. Leptospirosis

Leptospirosis, or Weil’s disease, is a zoonotic disease caused by Gram-negative spirochetes (*Leptospira* spp.). It is spread by exposure to contaminated fresh water, and is most prevalent in areas where sanitation is poor. Rodents are important reservoirs, and they maintain the transmission cycle. Leptospirosis can be found worldwide, but is most prevalent in Southeast Asia, East-Central Africa, and Oceania, in tropical, subtropical or coastal climates. The incubation period is typically 2 to 30 days; however, most patients develop symptoms 7 to 12 days after exposure [5,40,41].

Initial symptoms resemble a viral illness: acute fever, malaise, myalgia, predominately in the calves, conjunctival suffusion, and, occasionally, vomiting or diarrhea. In its most severe form it can be a multisystem disease with some patients jaundiced and with acute renal failure, and even with hemorrhagic complications, especially pulmonary [3,5,40,41]. Empirical treatment should be initiated early, with doxycycline or azithromycine, and in cases of severe illness, intravenous ceftriaxone, penicillin G or doxycycline [41].

For travelers or others at high risk (for example, people engaging in water sports or field work), prophylaxis with oral doxycycline 200 mg once a week may be considered, although evidence of its protective efficacy is limited and data remain mixed [42].

#### 3.3.2. Typhoid Fever

Typhoid fever is a systemic infection caused by the Gram-negative bacterium *Salmonella enterica* serotype Typhi. Transmission is fecal–oral, through ingestion of food or water contaminated with human feces [2,3,15].

The disease is endemic to parts of South Asia (India and Pakistan) and Southeast Asia, and sub-Saharan Africa. In Europe and North America, the vast majority of cases diagnosed by doctors occur in returning travelers, with the highest risk in those visiting friends and relatives. The incubation period varies from 6 to 30 days.

Typhoid fever can present in a variety of ways, and its symptoms frequently mimic those of other febrile illnesses like malaria. The usual symptom complex includes sustained high fever, headache, malaise, nausea, abdominal pain, and either constipation or diarrhea. In severe cases, the disease can cause sepsis, intestinal hemorrhage or perforation, and neurological complications. Some people can also become asymptomatic chronic carriers, representing an infectious risk to others [2,3,15,43,44].

Diagnosis is based on microbiologic confirmation. Blood cultures are the golden standard and are most sensitive in the first week of the illness. Stool cultures become more sensitive later in the course of disease. PCR testing remains positive longer than cultures, stains and rapid antigen tests [2,3,15].

Treatment has been complicated by regional antimicrobial resistance. First-line agents such as ampicillin and trimethoprim–sulfamethoxazole are ineffective against multidrug-resistant (MDR) strains, particularly those contracted in South Asia and sub-Saharan Africa. Also, resistance to fluoroquinolones is now very common in Southeast Asia. Current guidelines advocate the use of azithromycin or third-generation cephalosporins as empirical therapy, with carbapenems reserved for use with extensively drug-resistant infections [43,44].

Vaccination against typhoid fever is recommended for travelers to endemic areas. However, the currently available vaccines offer only partial protection, and should be supplemented by careful attention to food and water hygiene [43,44].

#### 3.3.3. Malaria

As described in the section above.

#### 3.3.4. Q Fever

Q fever is a zoonosis caused by the Gram-negative, obligate intracellular bacterium, *Coxiella burnetii*. The disease is found worldwide in rural areas where certain livestock—specifically, cattle, sheep, and goats—are present, with New Zealand being an exception. Infection is mainly by inhalation of infected animal birth products, urine, feces or dust [39].

Acute infection is often silent or relatively mild and nonspecific, presenting with fever, headache, myalgia, and sometimes pneumonia or hepatitis. In contrast, chronic infection is marked with worse sequelae, including death. Chronic infection is rare, and is most frequently diagnosed in the form of endocarditis.

Diagnosis is made based on serology and PCR. Blood cultures are usually negative, presumably because the organism is almost exclusively intracellular. Doxycycline is also effective in treating acute Q fever in children and adults. Chronic infection is treated with prolonged combination therapy with at least two antibiotics, most commonly doxycycline and hydroxychloroquine [39,45].

#### 3.3.5. Brucellosis

Brucellosis is a systemic zoonotic infection due to Gram-negative coccobacilli of the genus *Brucella*. The disease is endemic in areas with large livestock industries and poor animal vaccination, especially in the Mediterranean basin, the Middle East, Central Asia, sub-Saharan Africa and parts of Latin America.

Infection occurs most frequently through direct contact with infected animals or consumption of unpasteurized dairy products, but another potential route of exposure is from contaminated aerosols.

Clinical symptoms may be variable and with nonspecific features, but patients frequently present with prolonged febrile illness characterized by foul-smelling sweats, malaise, arthralgia, myalgia, weight loss and hepatosplenomegaly, and it can also lead to conditions including osteoarticular disease, endocarditis, neurobrucellosis, and genitourinary infection.

Positive serology is needed for diagnosis.

Treatment requires prolonged combination antibiotic therapy, usually with doxycycline and rifampicin, for at least six weeks. In more severe or focal disease, doxycycline is combined with an aminoglycoside [46].

### 3.4. Long Incubation Time (>30 Days)

#### 3.4.1. Schistosomiasis

Schistosomiasis, a parasitic disease caused by infection with trematodes of the genus Schistosoma, is prevalent in many parts of the world, with more than 90% of cases occurring in Africa and additional endemic areas in South America and Southeast Asia. Infection occurs after contact with infected freshwater The incubation period is usually three to six weeks.

Schistosomiasis infections are often first manifest by local dermatitis at the point of inoculation of the cercariae (“swimmer’s itch”). However, patients develop acute schistosomiasis, also known as Katayama fever, two to nine weeks after exposure to fresh water, when the host immune response to the migration and maturation of the larvae increases. Patients typically have a syndrome characterized by fever, urticaria or rash, cough and wheezing, myalgias, abdominal pain, diarrhea, vomiting, and marked eosinophilia. Hepatosplenomegaly and pulmonary infiltrates are often present. Chronic infection with schistosomes occurs when eggs are laid in the intestines or the genitourinary tract [47,48].

*S. mansoni,* which is found in Africa, the Middle East and the Caribbean, and *S. japonicum*, which is found in China, the Philippines and Indonesia, infect the intestines and cause intestinal disease. *S. haematobium*, which is found in Africa and the Middle East, infects the genitourinary tract and causes genitourinary disease.

Eosinophilia is a common early finding, and serology directed against the pathogen may be positive in the acute phase of infection. However, the standard diagnostic method used is microscopic detection of eggs in the stool or urine, which depends on egg production and is typically undertaken 30 to 50 days after exposure, in order to ensure adequate sensitivity. In cases of suspected acute schistosomiasis, diagnosis with serology and microscopy is often insensitive, and empirical treatment may be necessary.

Prazinquantel is the treatment of choice, at a dose of 40 mg/kg in a single dose for most species but 60 mg/kg in three doses for *S. japonicum*. Treated has to be repeated after six to eight weeks because eggs and immature schistosomula can resist the initial treatment. Therapy for acute schistosomiasis can actually worsen symptoms, due to an allergic response to dying worms, and this occurs in about 50 percent of patients treated with praziquantel.

Therefore, corticosteroids are increasingly recommended to mitigate the inflammatory response of acute schistosomiasis, even before antiparasitic treatment, which is often started at 8 weeks after infection, to avoid the allergic response. The only means of prevention is to keep away from infected freshwater, since even the strongest insect repellent does not protect against schistosomiasis [48].

#### 3.4.2. Leishmaniasis

Leishmaniasis is a vector-borne parasitic disease caused by protozoa of the genus *Leishmania,* which are transmitted to people by the bites of infected female sandflies. The disease is endemic in parts of Africa, the Middle East, Central and South America, the Indian subcontinent, and the Mediterranean basin, with tropical and sub-tropical regions being the most affected.

The disease has a spectrum of clinical manifestations and, classically, has three major clinical syndromes: cutaneous, mucocutaneous, and visceral leishmaniasis. Cutaneous disease usually manifests as one or more painless ulcers or nodules at the site of inoculation by the sandfly or its bite. These may last for months and heal with scarring [49].

Mucocutaneous leishmaniasis is most often due to *L. braziliensis* and causes destructive lesions of the nasal, oral and pharyngeal mucosa.

Visceral leishmaniasis (kala-azar), caused mainly by *L. donovani* and *L. infantum*, is a severe systemic illness characterized by prolonged fever, weight loss, massive hepatosplenomegaly, pancytopenia, and hypergammaglobulinemia, and it is nearly always fatal if untreated.

Visceral leishmaniasis is the only one that presents with fever.

Thorough history taking is key, as symptoms may only appear months or years after infection.

Diagnosis is based on demonstration of the parasite in tissue specimens (e.g., skin lesion aspirates, bone marrow aspirates, spleen biopsies, lymph node biopsies), usually done by microscopy, isolation in culture, or PCR.

Serologic testing is useful for diagnosis of visceral disease, but is less reliably applied to cutaneous forms.

Liposomal amphotericin B is first-line therapy for visceral leishmaniasis in many parts of the world. Good responses also occur with other agents such as miltefosine, paromomycin, and various forms of amphotericin B [50].

#### 3.4.3. Malaria

As described in the section above.

### 3.5. Most Frequent Travel-Related Febrile Diseases by Endemic Region in Order of Frequency

#### 3.5.1. Sub-Saharan Africa

- Malaria (P-falciparum).
- Dengue.
- Rickettsiosis.
- Other arboviruses (yellow fever).
- Viral hemorrhagic fevers.

#### 3.5.2. Southeast Asia

- Dengue.
- Rickettsiosis.
- Malaria (*P. vivax* more common).
- Chikungunya.
- Leptospirosis.
- Enteric fever (typhoid).

#### 3.5.3. South Asia

- Typhoid fever.
- Malaria.
- Dengue.
- Chikungunya.
- Visceral leishmaniasis.
- Arboviruses.

#### 3.5.4. Central America and Caribbean

- Dengue.
- Malaria.
- Chikungunya.
- Zika.
- Leptospirosis.

#### 3.5.5. Latin America

- Dengue.
- Malaria.
- Leptospirosis.
- Yellow fever.
- Chagas disease.
- Rickettsiosis.

#### 3.5.6. Middle East

- Dengue.
- Rickettsiosis.
- Leishmaniasis.
- Viral hemorrhagic fever.

#### 3.5.7. Northern Australia

- Dengue.
- Rickettsiosis (tropical regions).
- Leptospirosis (wet season).

#### 3.5.8. North America

- Rickettsiosis (Rocky Mountain spotted fever).
- Lyme disease.

#### 3.5.9. Europe

- Rickettsiosis.
- Tick-borne diseases (Lyme, tick-borne encephalitis).

## 4. Discussion

This article and overview, e.g. Appendix A and the tables, is intended to provide a practical approach to the most frequent tropical infectious causes of fevers in returning travelers. It is not designed to be comprehensive, but rather to highlight diagnoses that must be considered in the emergency department. Per endemic region a different table is listed.

We aimed to develop a practical tool for the initial assessment, diagnostic workup, and empiric management of otherwise hemodynamically stable returning travelers with fever.

Diagnosing symptomatic returning travelers is difficult, because the differential diagnosis is broad, and many of the tests that must be ordered are time-consuming to perform. The history should include not just where someone traveled and the duration, but also when, and whether they were exposed to fresh water, animals, sexual contact, or any prophylactic measures. Most post-travel fevers are actually due to infections found in the home environment, including viral respiratory infections. While (recent) travel is still the most obvious explanation, these cannot be discounted as early diagnoses. This overview is designed to support E.D. decision making by including incubation periods, epidemiology, and clinical signs as factors to take into account. It is intended to be used by clinicians to clarify key decisions.

Also, we acknowledge that E.D. clinicians and other acute-care providers are often at a disadvantage when it comes to diagnosing and managing tropical infections. Clinicians should therefore seek early specialist input if there is diagnostic uncertainty or severe disease, in order to guide timely, appropriate management. The summary in Appendix A is intended for use during the initial consultation with the stable traveler. The appendix should be used in conjunction with local protocols and up-to-date epidemiological data. Our goal was to provide emergency physicians with a succinct, clinically relevant summary of diagnostic considerations and initial therapeutic approaches to the most important infectious diseases encountered in everyday practice. In developing this narrative review, we assembled an initial list of the most important infectious diseases encountered in these patients. Our intention was not to provide a systematic review, evidence-based diagnostic accuracy estimates, or a formally validated clinical prediction model.

Rather, the aim of our paper is decidedly pragmatic. We sought to distill the existing literature into a practical framework to support front-line clinicians in making sensible decisions during the work-up of pericarditis.

Future systematic reviews could add considerable new value by including quantitative analyses of diagnostic performance, wider searches of the literature base, and formal evaluation of the risk of bias. With a broader evidence base, these efforts may even provide the opportunity to generate validated diagnostic algorithms and evidence-based clinical decision tools for the ED.

## 5. Limitations

This review has several significant limitations. While the literature search was structured, this review was narrative instead of systematic, and the sampling of literature was not guided by PRISMA or other formal quality-assessment guidelines or inclusion/exclusion criteria. Thus, selection bias is a concern. Although the literature search was executed in a structured manner, the potential for selection bias cannot be disregarded.

No formal statistical analyses or meta-analyses of diagnostic accuracy were performed. Thus, our approach to diagnosing myocarditis should not be considered a validated clinical decision rule, and no conclusions can be drawn about the sensitivity, specificity, predictive values, or overall diagnostic performance of our clinical approach.

We do agree that systematic reviews and studies of diagnostic accuracy would be an important next step. Our goal was to provide a practical, clinically useful overview for emergency physicians involved in the work-up of the febrile returning traveler. Our manuscript provides an accessible synthesis of the available literature that is applicable to day-to-day clinical practice, although the level of evidence cited is necessarily pragmatic.

Formal risk-of-bias assessments should also be used in the future. As the evidence base on this topic is continuing to expand, future systematic reviews should also include broader database searches and quantitative analyses of diagnostic performance, which may lead to the development of validated diagnostic algorithms and evidence-based clinical decision tools specific to the emergency department.

## 6. Conclusions

Fever in returning travelers is a relatively common and challenging problem in emergency departments.

In non-endemic settings, ED clinicians are often unfamiliar with tropical diseases, due to limited exposure. Although a wide range of exotic infections exist, the conditions discussed in this article represent the most common causes of travel-related fever, and are the most relevant for initial ED assessment and management. This list is not exhaustive, and other (imported or cosmopolitan) infections that are less common may also cause fever after travel. Moreover, different infections can co-occur and may require different treatment (e.g., malaria, typhoid fever). Therefore, vigilance remains warranted after the initial evaluation, and additional clinical or biochemical clues should prompt further investigation whenever necessary.

The primary goal of the article is to provide clinicians with a practical, structured algorithm to better direct the early assessment and initial approach to hemodynamically stable travelers presenting with fever. The overview incorporates incubation periods, epidemiology, and unique clinical attributes, to allow clinicians to rapidly differentiate.

## Data Availability

No new data was generated for this study.

## Abbreviations

The following abbreviations are used in this manuscript:

ED	Emergency department
STI	sexually transmitted infections
VFR	visiting friends and relatives
ACT	artemisinin-based combination therapy
DENV	Dengue virus
DHF	Dengue hemorrhagic fever
DSS	Dengue shock syndrome
NSAIDs	non-steroidal anti-inflammatory drugs
ADE	antibody-dependent enhancement
CHIKV	Chikungunya virus
SFG	Spotted Fever Group
STG	Scrub Typhus Group
TG	Typhus Group
MDR	Multidrug-resistant

## Appendix A

**Table A1:** Sub-Saharan Africa.

Disease	Incubation Time	Key Symptoms	Suggestive Lab Abnormalities	Diagnosis	Treatment
Malaria	7–14 days	Fever, chills, general malaise	Anemia	Blood smear	ACT
Dengue	4–7 days	High fever, myalgia, rash	Thrombocytopenia	PCR/serology	Supportive care. No NSAIDs
Rickettsiosis	5–14 days	Fever, eschar, rash		PCR/serology	Doxycycline
Viral hemorrhagic fever	2–21 days	Fever, petechia, purpura, shock		PCR/serology	Isolation/supportive care

**Table A2:** Southeast Asia.

Disease	Incubation Time	Key Symptoms	Suggestive Lab Abnormalities	Diagnosis	Treatment
Dengue	4–7 days	High fever, myalgia, rash	Thrombocytopenia	PCR/serology	Supportive care. No NSAIDs
Rickettsiosis	5–14 days	Fever, eschar, rash		PCR/serology	Doxycycline
Malaria (*P. vivax*)	7–30 days	Fever, chills, general malaise	Anemia	Blood smear	ACT + primaquine
Chikungunya	3–7 days	Fever, severe (symmetric) arthralgia		PCR/serology	Supportive care
Leptospirosis	2–30 days	Fever, calf pain, water exposure, jaundice	Elevated bilirubin	PCR/serology	Doxycycline/Ceftriaxone
Typhoid fever	6–30 days	Sustained fever, GI complaints		Blood culture	Ceftriaxone/Azithromycin

**Table A3:** South Asia.

Disease	Incubation Time	Key Symptoms	Suggestive Lab Abnormalities	Diagnosis	Treatment
Typhoid fever	6–30 days	Sustained fever, GI complaints		Blood culture	Ceftriaxone/Azithromycin
Malaria	7–14 days	Fever, chills, general malaise	Anemia	Blood smear	ACT
Dengue	4–7 days	High fever, myalgia, rash	Thrombocytopenia	PCR/serology	Supportive care. No NSAIDs
Chikungunya	3–7 days	Fever, severe (symmetric) arthralgia		PCR/serology	Supportive care
Leishmaniasis	Weeks–months	Fever, splenomegaly, ulcer		PCR/serology	Liposomal Ampho B

**Table A4:** Central America and Caribbean.

Disease	Incubation Time	Key Symptoms	Suggestive Lab Abnormalities	Diagnosis	Treatment
Dengue	4–7 days	High fever, myalgia, rash	Thrombocytopenia	PCR/serology	Supportive care. No NSAIDs
Malaria	7–14 days	Fever, chills, general malaise	Anemia	Blood smear	ACT
Chikungunya	3–7 days	Fever, severe (symmetric) arthralgia		PCR/serology	Supportive care
Zika	3–14 days	Mild fever, conjunctivitis		PCR/serology	Supportive care
Leptospirosis	2–30 days	Fever, calf pain, water exposure, jaundice	Elevated bilirubin	PCR/serology	Doxycycline/Ceftriaxone

**Table A5:** Latin America.

Disease	Incubation Time	Key Symptoms	Suggestive Lab Abnormalities	Diagnosis	Treatment
Dengue	4–7 days	High fever, myalgia, rash	Thrombocytopenia	PCR/serology	Supportive care. No NSAIDs
Malaria	7–14 days	Fever, chills, general malaise	Anemia	Blood smear	ACT
Leptospirosis	2–30 days	Fever, calf pain, water exposure, jaundice	Elevated bilirubin	PCR/serology	Doxycycline/Ceftriaxone
Rickettsiosis	5–14 days	Fever, eschar, rash		PCR/serology	Doxycycline

**Table A6:** Middle East.

Disease	Incubation Time	Key Symptoms	Suggestive Lab Abnormalities	Diagnosis	Treatment
Dengue	4–7 days	High fever, myalgia, rash	Thrombocytopenia	PCR/serology	Supportive care. No NSAIDs
Rickettsiosis	5–14 days	Fever, eschar, rash		PCR serology	Doxycycline
Schistosomiasis	3–6 weeks	Katayama fever, rash	Eosinophilia	Serology/urine	Praziquantel
Leishmaniasis	Weeks–months	Fever, splenomegaly, ulcer		PCR/serology	Liposomal Ampho B
Viral hemorrhagic fever	2–21 days	Fever, petechia, purpura, shock		PCR/serology	Isolation/supportive care

**Table A7:** Northern Australia.

Disease	Incubation Time	Key Symptoms	Suggestive Lab Abnormalities	Diagnosis	Treatment
Dengue	4–7 days	High fever, myalgia, rash	Thrombocytopenia	PCR/serology	Supportive care. No NSAIDs
Rickettsiosis	5–14 days	Fever, eschar, rash		PCR/serology	Doxycycline
Leptospirosis	2–30 days	Fever, calf pain, water exposure, jaundice	Elevated bilirubin	PCR/serology	Doxycycline/Ceftriaxone

**Table A8:** North America.

Disease	Incubation Time	Key Symptoms	Suggestive Lab Abnormalities	Diagnosis	Treatment
Rickettsiosis	5–14 days	Fever, eschar, rash		PCR/serology	Doxycycline
Lyme disease	3–30 days	Erythema migrans		Serology	Doxycyline
